# A Density-Based Multilevel Terrain-Adaptive Noise Removal Method for ICESat-2 Photon-Counting Data

**DOI:** 10.3390/s23249742

**Published:** 2023-12-10

**Authors:** Longyu Wang, Xuqing Zhang, Ying Zhang, Feng Chen, Songya Dang, Tao Sun

**Affiliations:** College of Geo-Exploration Science and Technology, Jilin University, Changchun 130026, China; longyu22@mails.jlu.edu.cn (L.W.); zxq@jlu.edu.cn (X.Z.); fchen21@mails.jlu.edu.cn (F.C.); dangsy22@mails.jlu.edu.cn (S.D.); suntao22@mails.jlu.edu.cn (T.S.)

**Keywords:** Ice, Cloud, and Land Elevation Satellite-2 (ICESat-2), terrain adaptation, photon-counting data noise removal, ordering points to identify the clustering structure (OPTICS)

## Abstract

The photon point clouds collected by the high-sensitivity single-photon detector on the Ice, Cloud, and Land Elevation Satellite-2 (ICESat-2) are utilized in various applications. However, the discretely distributed noise among the signal photons greatly increases the difficulty of signal extraction, especially the edge noise adjacent to signals. To detect signal photons from vegetation coverage areas at different slopes, this paper proposes a density-based multilevel terrain-adaptive noise removal method (MTANR) that identifies noise in a coarse-to-fine strategy based on the distribution of noise photons and is evaluated with high-precision airborne LiDAR data. First, the histogram-based successive denoising method was used as a coarse denoising process to remove distant noise and part of the sparse noise, thereby increasing the fault tolerance of the subsequent steps. Second, a rotatable ellipse that adaptively corrects the direction and shape based on the slope was utilized to search for the optimal filtering direction (OFD). Based on the direction, sparse noise removal was accomplished robustly using the Otsu’s method in conjunction with the ordering points to identify the clustering structure (OPTICS) and provide a nearly noise-free environment for edge searching. Finally, the edge noise was removed by near-ground edge searching, and the signal photons were better preserved by the surface lines. The proposed MTANR was validated in four typical experimental areas: two in Baishan, China, and two in Taranaki, New Zealand. A comparison was made with three other representative methods, namely differential, regressive, and Gaussian adaptive nearest neighbor (DRAGANN), used in ATL08 products, local distance statistics (LDS), and horizontal ellipse-based OPTICS. The results demonstrated that the values of the F1 score for the signal photon identification achieved by the proposed MTANR were 0.9762, 0.9857, 0.9839, and 0.9534, respectively, which were higher than those of the other methods mentioned above. In addition, the qualitative and quantitative results demonstrated that MTANR outperformed in scenes with steep slopes, abrupt terrain changes, and uneven vegetation coverage.

## 1. Introduction

Spaceborne LiDAR has been widely used in global forest canopy height estimation [1,2,3] due to its rapid and cost-effective detection of surface typography and vertical forest structure [2,4,5,6]. The accuracy of global forest height data evaluated by the Geoscience Laser Altimeter System (GLAS) onboard the Ice, Cloud, and Land Elevation Satellite (ICESat) has been proven over the past few decades [7]. However, limitations such as a large footprint size (70 m) and low sampling density (along-track spacing of 170 m) restrict its application in complex terrains [8]. To address these issues, the ICESat-2, equipped with the advanced topographic laser altimeter system (ATLAS) [9], was launched and put into operation in September 2018. In contrast to the full waveform LiDAR system from the GLAS, ATLAS is a photon-counting LiDAR that utilizes high-frequency pulses [10] with an emission energy of 160/40 μj at 10 kHz and 0.7 m along-track resolution for precise surface measurements [11,12], providing a high sampling rate, a small footprint size (10 m), and a nearly continuous along-track strip of point cloud data [13]. However, the original data from ATLAS, particularly the daytime data, are heavily contaminated by background noise arising from solar radiation and dark current returns [14] and feature a random and wide distribution. In vegetation coverage areas, the abundant noise distributed adjacent to the canopy and the ground surface poses significant challenges for identifying the canopy and ground signals in photon-counting LiDAR data. In this sense, signal photon discrimination from the original data is necessary to address these challenges.

Many excellent signal detection algorithms for photon point clouds have been developed. The algorithms can be broadly classified into three categories: grid-based two-dimensional image processing techniques, local parameters statistics, and density-based clustering algorithms [15]. For image-based techniques, the rasterization of point clouds tends to lead to poor accuracy, so dominant studies mainly focus on the last two types. Local statistical methods separate signals from noise by constructing point cloud features, followed by global or local thresholds. The selection of thresholds in such methods influences the resulting measurements, such as DRAGANN [16,17,18] used in ATL08 products and local distance statistics (LDS) [19]. Density clustering methods utilize a priori information that ICESat-2 point clouds are densely concentrated in the signal photons in comparison to the noise photons for denoising purposes. This type of method imposes no requirements on the shape of clusters [20], but it is sensitive to the parameter settings, such as Bayesian [21], dense-based spatial clustering of applications with noise (DBSCAN) [22], and OPTICS [23]. Notably, OPTICS does not generate explicit clusters and is more robust than the other clustering approaches.

At present, most improvements towards the algorithms above [16,19,20,21,22,23] mainly focus on four aspects: improving the search domain, enhancing parameter determination, employing several characteristics with machine learning methods, and combining several single-level methods to achieve better accuracy. When it comes to search domain improvement, horizontal ellipse-based OPTICS was developed by Zhu et al. [24], which demonstrated greater robustness than the optimized DBSCAN [24]. Gao et al. [25] enhanced the DRAGANN algorithm with a multi-scale search radius for solving the residual noise caused by a fixed radius. In terms of parameter determination, Huang et al. [26] and Zhang et al. [27] improved the self-adaptability of density-based methods based on a genetic algorithm and a particle swarm algorithm. Li et al. [28] and He et al. [29] proposed a relative neighboring relationship (RNR) and local outlier factor algorithm with a rotating search area (LOFR) to improve the local parameter statistics algorithm. For supervised machine learning techniques, Li et al. [30] and Chen et al. [31] proposed the neighboring forward local density difference (NFLDD) and K-nearest neighbors distance to characterize the differences between the signals and noise. Supervised methods, such as random forest and support vector machine, were used to classify the photons, which achieved good accuracy, but these methods rely on a large number of training samples. There are no unified tools and standards for preparing samples, making it difficult apply them widely. However, there is residual noise after single-level filtering algorithms [24,25,26,27,28,29,30,31,32,33], especially noise adjacent to the signal in complex terrains.

Several multilevel approaches combined with single-level methods have been gradually developed. For example, Huang et al. [34] proposed a multilevel strategy that combined an adaptive filter TS-SCABR, empirical mode decomposition, and percentile statistics method to extract the canopy-top. Wu et al. [35] created a multilevel technique that incorporated an RNR-DCM filter and a sliding detection window and fitted a straight-line model to accurately distinguish the sub-canopy photons. To effectively achieve daytime data denoising, You et al. [36] proposed a hybrid algorithm that combined OPTICS and RNR-KNNB. These multi-level methods mainly include three steps, namely preprocessing, fine denoising, and residual noise processing [15], which usually focus on three problems: (1) Changing the traditional search domain to improve the adaptability and provide a stable denoising result in areas with complex terrains. (2) Constructing features or developing algorithms that increase the separability between the signal photons and the noise distributed around them by amplifying the differences between the two. (3) Exploring the adaptation of parameters for different data to make the algorithm more robust.

**Figure 1:**
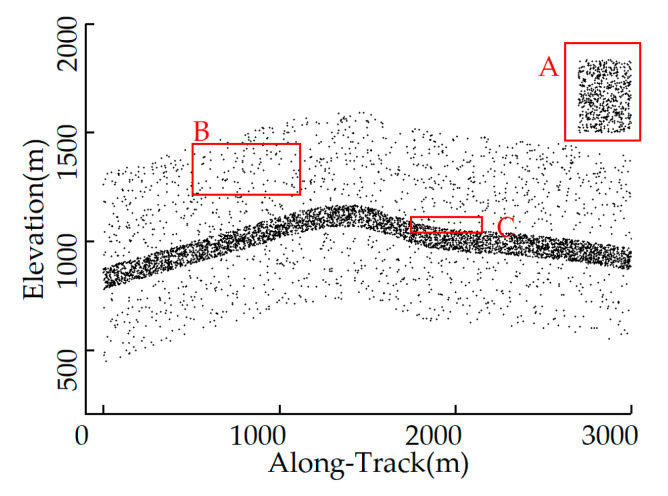
Schematic diagram of the noise distribution and types: A (cloud-caused high-altitude distant noise), B (sparse noise in medium distance), and C (edge noise adjacent to signal).

However, there are relatively few studies that consider all three factors above simultaneously. In this study, we propose a density-based multilevel terrain-adaptive noise removal method (MTANR) with a coarse-to-fine denoising strategy to address the issue. It is challenging to remove the noise adjacent to the signal and the removal requires a nearly noise-free environment; therefore, we categorized noise into three types [25,30]: cloud-caused high-altitude distant noise, sparse noise in medium distance, and edge noise adjacent to signal, as shown in Figure 1. Based on the classification of noise photons, the denoising process was divided into three steps: (1) Distant noise and part of sparse noise were removed using a histogram-based successive method which gradually tightened conditions as a coarse denoising process. (2) The residual sparse noise was identified by the terrain-adaptive OPTICS with a rotating search domain, and a nearly noise-free environment was provided. (3) Based on the directional distribution of local densities at the internal and boundary points in photon clusters, edge searching was employed to remove the edge noise, followed by surface line constraints, which further preserved the signal photons. The steps (2) and (3) are called fine denoising.

The main contributions of this study are as follows: (1) The proposed MTANR robustly preserves the signal photons in steep areas, abrupt terrain changes, and uneven vegetation coverage by automatically determining the terrain-based filtering direction and alternating the shapes of the search domain. (2) Edge searching improves edge noise removal capabilities and reduces edge noise’s impacts on ICESat2 data applications such as canopy height inversion.

The paper is organized as follows. In Section 2, the experiment and validation data are described. The details of the method are presented in Section 3. Experiments with four datasets are presented in Section 4 where the experimental results and analyses are presented. This is followed by some concluding remarks.

## 2. Materials

### 2.1. ICESat2 Data

**Figure 2:**
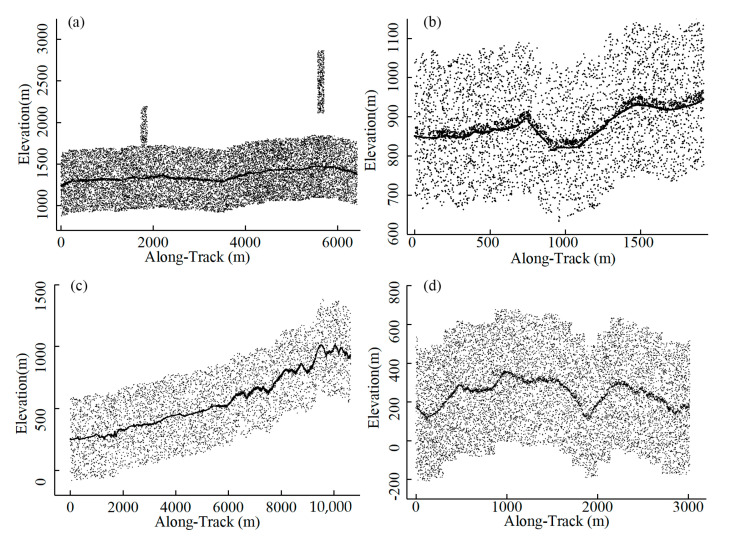
The raw ATL03 photon data: (**a**,**b**) in Baishan, China, and (**c**,**d**) in New Zealand.

The ATL03 photons and ATL08 product used for this study are accessible from the National Snow and Ice Data Center (https://nsidc.org/data/icesat-2/data, accessed on 4 March 2023), including information such as the signal confidence, elevation, latitude, and longitude. Considering the impacts of slope and vegetation coverage factors on denoising, this study opted to test two representative vegetation coverage study areas [37]. The profiles of the raw data selected are shown in Figure 2, and the details are displayed in Table 1. One study area is located in the vegetation coverage region near the southern foothills of the Changbai Mountain in Baishan City, Jilin Province, China. This area is characterized by medium to high and uneven vegetation. The other experimental region is situated in the Taranaki region of New Zealand, with relatively sparse vegetation and complex terrains. In this area, we selected two ATL03 strips with a total length of approximately 13 km. Figure 2c represents a strip extending from the mountain foot to the summit, covering gentle slopes, mountain peaks, and abrupt terrain changes. Figure 2d depicts a mountainous area with steep terrain and rapid changes in the slope. These two study areas exhibit obvious slope variations, uneven vegetation coverage distribution, and different background noise patterns. These factors collectively challenge the algorithm’s robustness and the precision of noise reduction [37].

### 2.2. Validation Data

ATL03 provides an initial classification of photons as signal [38] or background. Additionally, ATL08 also provides classification labels, and the photons in ATL03 can be easily reversed and indexed using the identified parameters [16,17,18]. For more information, see Table A1 in Appendix A. However, they are not accurate enough to serve as references for complex topographies such as hilly and mountainous terrains. Some existing approaches for quantitative denoising output assessment typically rely on the manual interpretation of Google Earth satellite images to construct validation datasets [39,40]. In this sense, these subjectively labeled references may be imprecise. For this reason, we employed the airborne point cloud with high density as the reference data to evaluate the signal extraction algorithm presented in this work [39], which provides an increasingly objective benchmark to quantify denoising accuracy. For supplementary validation, the digital terrain model (DTM) and digital surface model (DSM) with a horizontal resolution of 1 m from the airborne LiDAR data of approximately 10 pts/m^2^ were used to serve as boundaries for the signal photons. A photon is labeled as a signal when its elevation is within the DTM and DSM, and considered as noise when surpassing the threshold. Using airborne data as a constraint reduces subjectivity compared to manual classification, promising robust characterization over vegetation coverage areas.

Although it is ideal that the acquisition time of validation data and ICESat2/ATL03 data should be closely matched, this is often not possible due to the significant cost and time involved in airborne LiDAR missions and manual field surveys [41]. In addition, if there are no major disasters such as earthquakes, landslides, or forest fires, the changes in surface elevation and canopy height due to natural variability and vegetation growth should be minimal [42]. Despite differences in collection time, airborne data continue to be the best option for validation data, which have been used in several recent relevant studies [36,41,43,44,45] such as references for satellite-based forest canopy height inversion. Therefore, it is reasonable to assume that the airborne data provide good references. The acquisition time between airborne and spaceborne data is less than one year in New Zealand, and the airborne data can serve as references directly. The airborne data in Baishan was collected in 2014, a few years ahead of ICESat 2’s acquisition time. So in this area, we set a 0.5 m buffer zone to mitigate the impact of changes over time, according to the research of Huang et al. [39].

## 3. Method

A density-based multilevel terrain-adaptive noise removal method was designed to remove the high background noise, especially the edge noise of ICESat-2 photon data in study areas with complex terrain and vegetation coverage.

**Figure 3:**
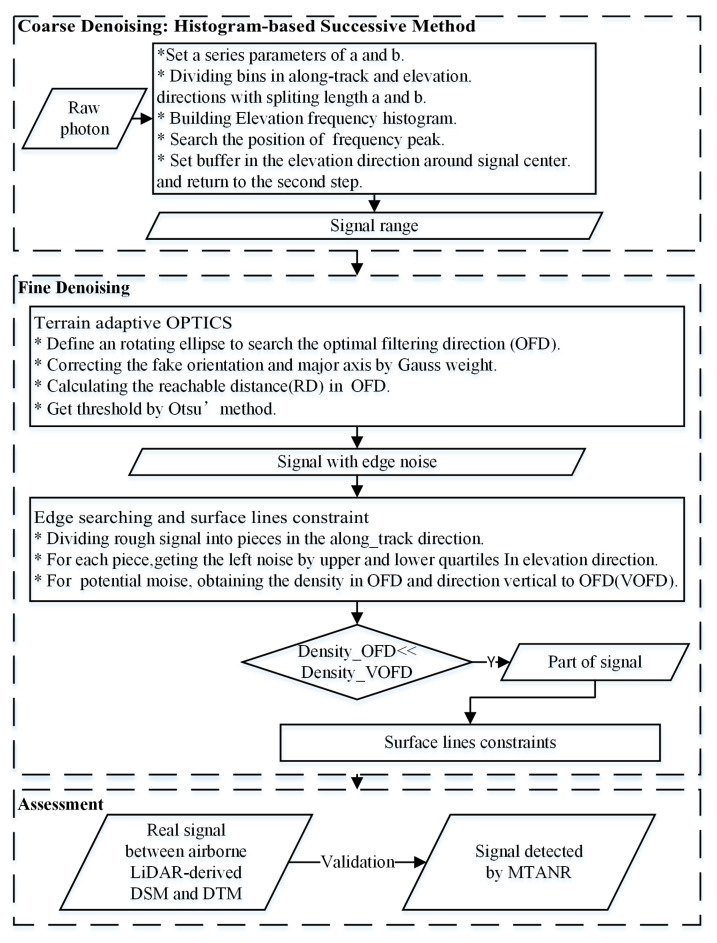
The flowchart of the noise removal algorithm.

As shown in the flowchart presented in Figure 3, different types of noise were removed using a coarse-to-fine strategy. First, the histogram-based successive denoising as a coarse denoising process was applied to remove distant noise and part of the sparse noise, reducing the computation in subsequent steps and increasing the fault tolerance in further denoising it to the maximum. Secondly, based on the coarse denoising result, fitted approximate terrain curves serve as an aid to the adaptive rotated ellipse to find the OFD. Then, the OPTICS algorithm was applied in the OFD to remove the residual sparse noise. Next, according to the directional distribution of local densities of the internal signal and edge noise in residual photon clusters, the OFD and the direction vertical to optimal filtering direction (VOFD) ellipses were used as search domains to characterize this distributional difference. As a result, the edge noise was removed well during the fine denoising process. In addition, surface lines were fitted to further preserve the signals between them. At last, the results were evaluated using the reference data from airborne LiDAR.

### 3.1. Preprocessing

**Figure 4:**
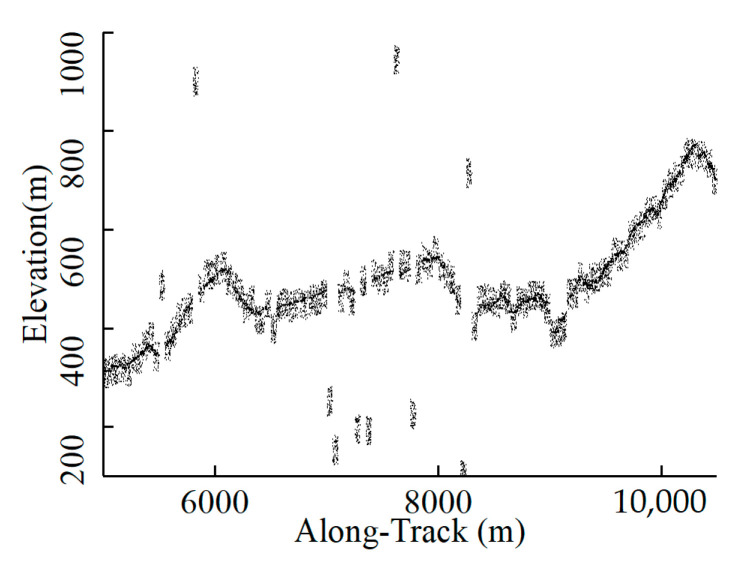
Schematic diagram: Misclassification of single-level histogram.

The main purpose of preprocessing is to remove cloud-caused distant noise and part of the sparse noise as a coarse denoising process. The cloud-caused noise clusters exhibit similar spatial distribution with signal photons, making it challenging to distinguish them from the signal at a global scale based solely on local density or distance features. Therefore, the handling of the residual portions will pose a challenge. Based on the significantly higher elevation of noise photons compared to signal photons, a histogram-based successive denoising method was designed to remove them. The specific steps of this method are as follows: (1) Grid partitioning: All photons were divided into several bins in the along-track and elevation directions with a certain step size. (2) Frequency histogram building: The number of photons in each bin was counted, and the signal centers were determined based on the elevation of the bin with the maximum quantity. To reduce the influence of individual extreme outliers, the signal center elevation was set as the arithmetic average of the median and the mean. (3) Buffer zone determination: A buffer zone was defined as the range of potential signal photons by taking a certain distance above and below the signal center. Figure 4 shows that by applying strict constraints, a single uniform split step size may identify the noise bins in the sky or below the ground as signals in areas with low surface coverage and sparse signal photons. Though using loose constraints may avoid this problem, our purpose of searching exact signal range will not be achieved. Above all, we devised a series of values, starting with a large partitioning interval and buffer, and successively tightening the parameters to restrict the signal photons within an increasingly fine range.

### 3.2. Terrain-Adaptive OPTICS Algorithm

OPTICS is an improvement in the DBSCAN algorithm. Consistent with the DBSCAN algorithm, it also has parameters such as neighborhood radius (Eps, ε) and minimum number of points in the neighborhood (MinPts). After giving the parameters, DBSCAN directly presents the clustering results. Compared to DBSCAN, the OPTICS algorithm does not directly output cluster assignments. Instead, it produces an ordered list of data points ranked by reachability distance (*RD*) in the hierarchical density-based cluster structure. By setting a threshold on *RD*, implicit clusters can be extracted. Thus, the cluster ordering from OPTICS is invariant to input parameters, while DBSCAN can produce various clusters with different parameters.

#### 3.2.1. Definition of Rotating Ellipsoidal OPTICS

Previous studies have found that ATL03 photons have a higher density in the along-track direction than in the across-track orientation [46]. To utilize this terrain-induced difference, we employed a rotating ellipsoidal search domain adapting to the local topography instead of the traditional circular neighborhood in OPTICS. For photon $p(x_{p},h_{p})$ and photon $q(x_{q},h_{q})$, the ellipsoidal distance between them is defined as Equation (1):

(1) $$\left\{ \begin{array}{l} \Delta a=x_{p}-x_{q},\Delta b=h_{p}-h_{q}\\ \mathrm{distance}(p,q)=\sqrt{{\left(\frac{\Delta a}{a}\right)}^{2}+{\left(\frac{\Delta b}{b}\right)}^{2}} \end{array}\right.$$

where Δa and Δb represent the along-track distance and the elevation between the two photons; *a* and *b* are the lengths of the major and minor axes of the search ellipse. When distance (p,q)<1, photon *q* is considered within the search ellipse centered at photon *p*; otherwise, photon *q* is outside the search ellipse. In areas of terrain relief, the search ellipse needs to be rotated to adapt to changes in the surfaces. In this case, the along-track and vertical distances are projected onto the major and minor axis directions of the rotated ellipse. The conversion method of Δa and Δb is illustrated in Equation (2), where *θ* is the counterclockwise angle between the major axis and the along-track direction.

(2) $$\left\{ \begin{array}{l} \Delta a=\cos\theta (x_{p}-x_{q})+\sin\theta (h_{p}-h_{q})\\ \Delta b=\cos\theta (h_{p}-h_{q})+\sin\theta (x_{p}-x_{q}) \end{array}\right.$$

In the OPTICS algorithm, ε-neighborhood, reachability distance (*RD*), and core distance (*CD*) are used to describe the relationship between photons, and here, we follow their definitions.

ε-neighborhood: For a point p in the full dataset *D*, its ε-neighborhood is defined as the set of points and manifested as $N_{\varepsilon }(o)$. It includes all data points in *D* that are within a distance ε away from the point *p*, which can also be represented as $N_{\varepsilon }(o)=\left\{x_{i}\in D|\mathrm{distance}(o,x_{i})\leq \varepsilon \right\}$. The number of samples contained in $N_{\varepsilon }(o)$ is denoted as $\left|N_{\varepsilon }(o)\right|$.

*CD*: Given ε and MinPts, the *CD* of a photon *o* can be expressed as Equation (3). When $|N_{\varepsilon }(o)|\geq \mathrm{MinPts}$, photon *o* is called the core point. In $N_{\varepsilon }(o)$, the *k*-th closest point to the photon *o* is denoted as $N_{\varepsilon }^{k}(o)$, and its *RD* can be marked as $\mathrm{dist}(o,N_{\varepsilon }^{MinPts}(o))$. If $\left|N_{\varepsilon }(o)\right|$ ≤ MinPts, CD(o) is identified as UNDEFINED.

(3) $$CD(o)=\left\{ \begin{array}{l} \mathrm{UNDEFINED},\;\;\;\;|N_{\varepsilon }(o)|<\mathrm{MinPts}\\ \mathrm{distance}(o,N_{\varepsilon }^{\mathrm{MinPts}}(o)),|N_{\varepsilon }(o)|\geq \mathrm{MinPts} \end{array}\right.$$

*RD*: As shown in Equation (4), if photon *o* is a core point, the *RD* of data point *p* to point *o* is defined as the maximum of CD(o) and distance(o,p). If photon *o* is not a core point, then RD(o) is determined as UNDEFINED.

(4) $$RD(o)=\left\{ \begin{array}{l} \mathrm{UNDEFINED},\;\;\;\;\;|N_{\varepsilon }(o)|<\mathrm{MinPts}\\ \max(CD(o),\mathrm{distance}(o,p)),|N_{\varepsilon }(o)|\geq \mathrm{MinPts} \end{array}\right.$$

#### 3.2.2. Optimal Filtering Direction Searching and Parameter Correction

**Figure 5:**
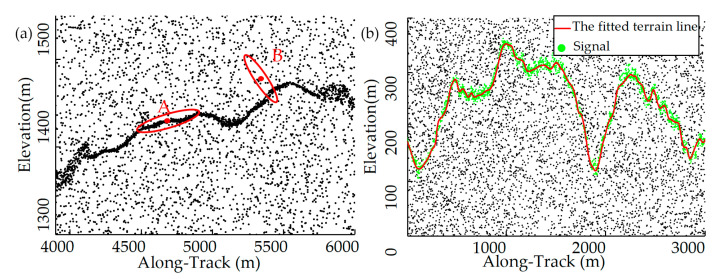
Schematic diagram: (**a**) false directions; (**b**) the fitted terrain line.

For a photon *p*, by changing the rotation angle *θ*, the number of neighborhood points in any direction and the OFD of photon *p* can be obtained (e.g., photon A in Figure 5a). To improve computational efficiency, the approximate direction was first searched at a step of 10°. Within a 10° range above and below this direction, the neighborhood counts were computed every 3°, and the OFD was determined as the direction with the maximum count. However, for near-ground edge noise photon *B* as shown in Figure 5a, this determination typically leads to searching in the wrong direction, decreasing the recognition rate for such photons.

(5) $$\left\{ \begin{array}{l} dist_{i}=\sqrt{{\left(x_{p}-x_{i}\right)}^{2}+{\left(h_{p}-h_{i}\right)}^{2}}\\ \sigma^{2}=\frac{\sum_{i=1}^{k}{\left(x_{i}-x_{p}\right)}^{2}+{\left(h_{i}-h_{p}\right)}^{2}}{k}\\ weight_{i}=1-\exp(\frac{-dist_{i}^{2}}{\sigma^{2}})\\ D=\sum_{i=1}^{k}dist_{i}\times weight_{i} \end{array}\right.$$

In this sense, we identified these photons and corrected false directions by defining local weighted density [27]. For any given point *p*, its density is assayed by Equation (5), where $dist_{i}$ represents the Euclidean distance between the *k*-th neighboring and the central photon, *k* denotes the number of photons in the neighborhood, $\sigma^{2}$ indicates the variance of the Gaussian function, $weight_{i}$ stands for the weight corresponding to the *i*-th neighborhood photon, and *D* is the density of the central photon.

The weight increases with the increasing distance between the neighborhood and central photons, and vice versa. This phenomenon significantly increases the density difference between signal and noise photons. By dividing the search ellipse into two parts based on the short axis, the densities were calculated separately for each part. When the density on one side is three times greater than the other, it is considered an excessive difference in neighboring densities. In such cases, the optimal filtering direction is considered false [47].

After a histogram-based successive coarse denoising, the signal centers along the trajectory were obtained. As shown in Figure 5b, a curve that roughly depicts the topography of this region was produced after sampling and fitting these signal centers. For photons detected in false directions, the wrong direction will be replaced by the tangent direction of the corresponding curve at the along-track distance during neighborhood search.

**Figure 6:**
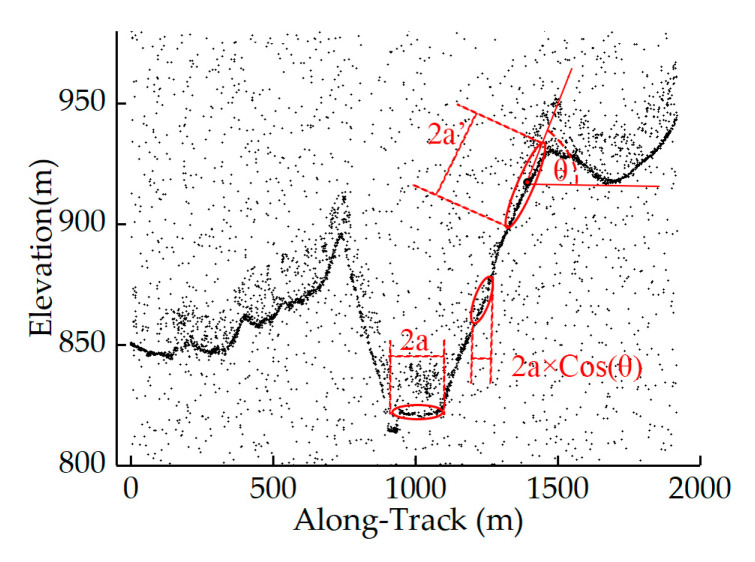
Schematic diagram: correction of search domain shape.

In addition, to ensure the consistency of the search ellipse in the along-track direction, the ellipse size needs to be modified based on the slope angle during the neighborhood search, considering that during the ground detection of ATLAS photons, the sensor collects point clouds over sloped and flat terrains with consistent firing and sampling rates. As shown in Figure 6, when the search direction exhibits an oblique angle to the ground, the along-track coverage of the ellipse will be shortened when using a fixed-size filtering kernel. Consequently, in sloped regions, a fixed search domain will therefore cover fewer pulses, thereby underestimating the photon density and influencing the denoising performance. Thus, the major axis of the search ellipse in sloped regions can be corrected by Equation (6), where *a′* is the major axis over the flat terrain, *θ* denotes the angle between the search and horizontal directions, and *a′* represents the major axis over the sloped terrain.

(6) $$a^{\prime }\times \cos(\theta )=a$$

Given that for a photon there may be multiple *RDs* for it, the minimum *RD* is set as the optimal *RD* for it. After computing the optimal *RDs* for all photons, a threshold was obtained using Otsu’s method [24,48,49,50]. Based on the sparse distribution of noise photons, we identified photons with *RDs* greater than the threshold as noise. Considering that noise densities may vary over segments, the photons were divided into 1000 m segments to mitigate this effect, and Otsu’s method was applied independently within each segment.

### 3.3. Edge Searching and Surface Lines Constraints

**Figure 7:**
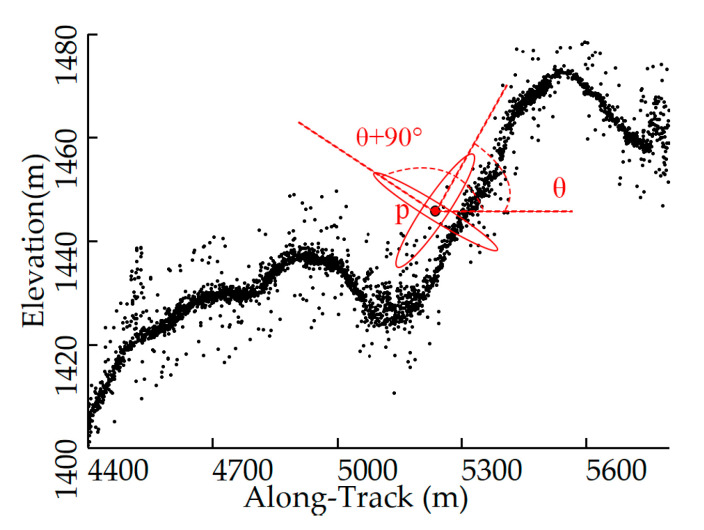
Schematic diagram: the principle of edge searching.

After the processing of histogram-based successive denoising and the terrain-adaptive OPTICS, most noise photons were removed. The remaining photons were considered to comprise only signal photons and edge noise. For the noise shown in Figure 7, the number of neighboring points for photon *p* is significantly lower in the OFD than in the VOFD. Based on these characteristics, a signal can be distinguished from the edge noise.

Within a local window, noise photons may exist in the elevation percentile ranges of 0–25% and 75–100%, which were selected to define potential noise across each along-track segment [25]. Next, iteration through these potential noise points was conducted. For each photon, we searched along and perpendicular to the OFD and compared the point counts in both directions. When the number in the VOFD is greater than that in the OFD, it is labeled noise. Otherwise, it is corrected to be signal. After this process, the remaining photons were all considered signals. However, the laser pulse may be reflected by branches and leaves, decreasing the number of photons penetrating the ground. Consequently, the understory points exhibit relatively sparse distribution and may be incorrectly clustered as noise. To preserve these photons, the highest and lowest points per segment were fitted after the previous process, the approximate DTM and DSM were fitted, and the photons with elevations between the fitted models were reserved. Both Section 3.2 and Section 3.3 contain the fine-denoising process.

### 3.4. Accuracy Assessment

In this paper, the performance of the algorithm on the ATL03 data is evaluated both qualitatively and quantitatively. For quantitative assessments, recall of recall (*R*), precision (*P*), and the value of F1 score (*F*) are adopted. In addition, the recall rate of signal (*R_s_*) and removal rate of noise (*R_n_*) are used to evaluate the performance of preprocessing. These accuracy metrics can be calculated by Equation (7).

As Table 2 shows, *TP* represents photons with true and predicted labels as signals; *FN* denotes photons with a true label as a signal but the predicted as a noise; *FP* implies photons with a true label as a noise but a predicted one as a signal; *TN* indicates photons with both true and predicted labels as noise.

For better qualitative assessment, type 1 error and type 2 error indicators are used to qualitatively evaluate the performance of different algorithms [51]. The type 1 error is used to indicate when noise photons are misclassified as signal photons, and the type 2 error indicator is used to indicate when signal photons are misclassified as noise photons.

(7) $$\left\{ \begin{array}{l} R_{s}=\frac{TP}{TP+FN}\\ R_{n}=\frac{TN}{TN+FP}\\ P=\frac{TP}{TP+FP}\\ F=\frac{2\times P\times R}{P+R} \end{array}\right.$$

## 4. Experiment Results and Analysis

### 4.1. Coarse Denoising: Histogram-Based Successive Denoising

**Figure 8:**
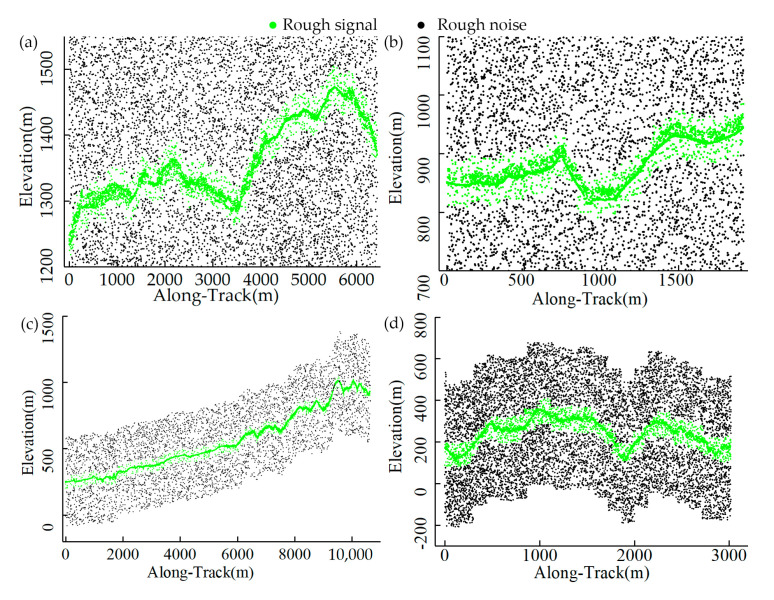
Results of the histogram-based successive denoising: (**a**) Data 1, (**b**) Data 2, (**c**) Data 3, and (**d**) Data 4.

The results of the four selected datasets subjected to histogram-based successive coarse denoising are shown in Figure 8. The distant noise and most sparse noise have been removed and the signal photons have been restricted to a range that is as accurate as possible, but the residual noise still needs further fine-tuning. Considering the purpose and nature of coarse denoising, for quantitative evaluation, we only compute the recall rate of signal and noise, which is shown in Table 3. As Table 3 shows, in four datasets, the *R_s_* values of the proposed coarse denoising method all reach 1 and the *R_n_* values are above 0.8, indicating that the method removes most of the noise photons and preserves all the signals.

### 4.2. Fine Denoising: Terrain-Adaptive OPTICS and Edge Searching

**Figure 9:**
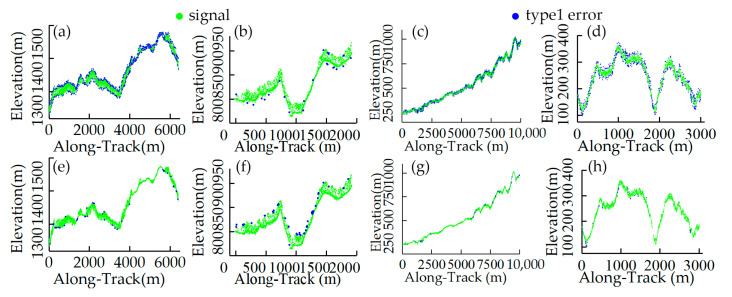
Results of fine denoising. (**a**–**d**) are the denoising results of adaptive OPTICS, and (**e**–**h**) are the denoising results after edge searching.

Fine denoising assignment, which consists of adaptive OPTICS, edge searching, and surface constraints, was utilized to remove the noise distributed around the signal, and the detailed results are shown in Figure 9.

From Figure 9a–d, adaptive OPTICS correctly detected almost all of the sparse noise and adapted well to the four datasets with different slopes. However, there were still some noise photons adjacent to the signal, especially in regions covered by sparse vegetation. Edge searching was applied to characterize the difference between edge noise and the spatial distribution of the signal, thereby allowing the removal of the noise, as shown in Figure 9e–h. The misclassified edge noise photons were almost identified by edge searching, and the change in accuracy metrics is shown in the last two columns of Table 4. From Table 4, it is clear that for terrain-adaptive OPTICS, the *F* values for four datasets are all above 0.94, but the *p* values for Data 1 and Data 4 are a little low due to the effect of the large amount of misclassified noise photons. After edge searching, the *F* values and *p* values of the four regions are improved to varying degrees due to the removal of most of the residual noise, although there is a slight decrease in the *R* values. For sparse noise photons, OPTICS does well in removing them, but it tends to fail in identifying the edge noise recognized by edge searching, which is consistent with the results of the visual analysis above.

### 4.3. Comparative Analysis of Several Methods in Typical Areas

**Figure 10:**
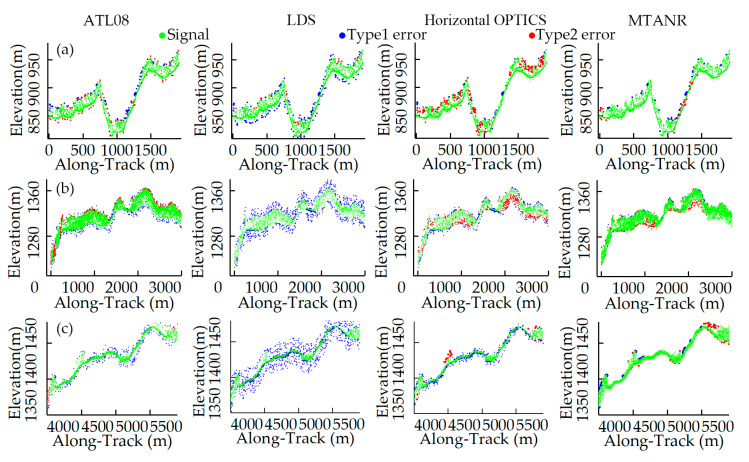
Zoom-in view of denoising results using different methods in Data 1 and Data 2: (**a**) areas covered by asymmetric vegetation, (**b**) areas covered by tall trees, and (**c**) low fraction vegetation coverage.

To better evaluate the performance of our algorithm, raw photons were also processed by ATL08, LDS, and the horizontal ellipse-based OPTICS [24] on the selected datasets. Figure 10 depicts the details of point clouds in Baishan based on different methods. In three typical situations, ATL08 and the MTANR demonstrate good denoising outputs, removing most of the noise photons while maximally retaining the signal photons. Although most signal photons are preserved through LDS, this method fails to identify the edge noise (e.g., type 1 error in Figure 10). The horizontal OPTICS is less affected by edge noise than the LDS. However, lots of sparse signal photons under the canopy (e.g., type 2 error in Figure 10a,b) are misidentified as noise, causing discontinuities in the ground surface. Apparently, these errors will greatly influence the extracted canopy height and DEM in this situation.

The reason for this phenomenon is that the vegetation cover is uneven under the influence of slopes. Flat areas with high fractional vegetation coverage are endowed with tall trees, and the vegetation in rugged areas is short and sparse. For photons beneath trees, canopy occlusion exerts varying effects depending on vegetation density. In sparse vegetation zones, the canopy has less blockage of laser pulses, leading to denser understory points. By contrast, lush vegetation areas with a denser canopy display sparser understory returns. These sparse photons differ slightly from noise. In rugged terrains, the horizontal elliptical search window cannot effectively leverage the terrain information, misclassifying the sparse ground photons. Based on a terrain-adaptive ellipse search window, the MTANR acclimates to the altered terrain, improving the extraction of signal photons in different slopes. Additionally, the edge searching and strengthened constraints for surface lines further preserve understory sparse photons and amplify the variability between photons and edge noise. From Table 4, the MTANR outperforms the other methods pronouncedly in terms of *p* and *F* in the experimental sites, which is consistent with Figure 10. Although LDS has the highest *R* values, it exhibits a poor ability to distinguish edge noise. To summarize, in a rugged and uneven vegetated area, our method is more suitable for signal extraction than the other three methods.

**Figure 11:**
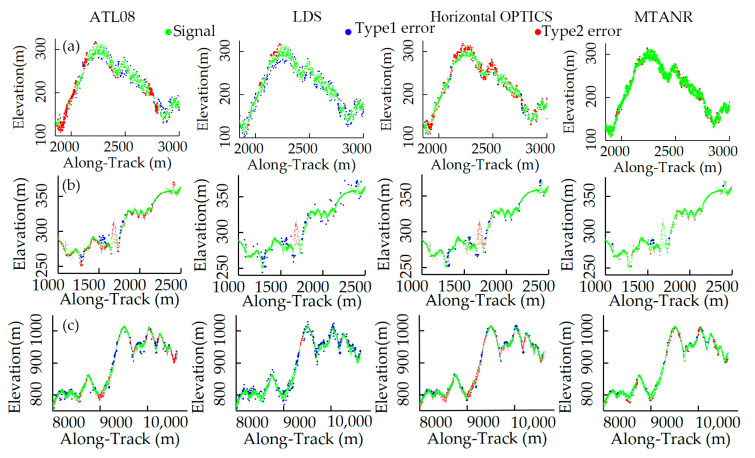
Zoom-in view of denoising results from different methods in Data 3 and Data 4: (**a**) steep areas, (**b**) terrain with abrupt changes, and (**c**) mountaintop.

Figure 11 presents a detailed comparison of denoising outputs from compared methods over mountain peaks, terrain with abrupt changes, and steep slopes. As shown in Figure 11a,b, ATL08 typically retains signal photons and misses sparse signals (type 2 error) across terrain discontinuities and steep slopes covered by short vegetation, resulting in discontinuous ground photons. Due to the limitations of horizontal ellipse search space, sparse signals in the two steep regions and abrupt changes in the terrain are misclassified as noise (e.g., type 2 error of Horizontal OPTICS in Figure 11) by horizontal OPTICS. LDS preserves more photons in this test region than ATL08 and horizontal OPTICS, avoiding sparse signal loss, but more edge noise photons (e.g., type 1 error in Figure 11) are also preserved. This phenomenon can be primarily attributed to two factors. For one thing, even with a high-frequency micro-pulse laser detecting height values at a resolution of 0.7 m, ICESat-2 only records 0–4 photons from a reflected laser pulse over vegetation. For another, ICESat-2 has consistent pulse frequencies and sampling rates over surfaces with different slopes. For the same distance along the track, there are fewer returning photons in steep terrain than in flat areas with the slopes increasing [47,52]. It is necessary to expand the search domain to contain more photons for canopy detection. Therefore, there is a reasonable chance that the signals are inadequately characterized by the fixed search domain [53], accounting for the interrupted signal segments in the ATL08 and horizontal OPTICS denoising results, as shown in Figure 11a. According to the quantitative metrics in Table 4, LDS outperforms ATL08 in the *R* values, but its *p* values are significantly lower than the other methods. In both areas, the MTANR balances the *R* and *p* values and exhibits higher *F* values than the prior methods. Overall, in areas with steep slopes and sparse vegetation cover, this method exerts the best denoising performance among these approaches and is advantageous in detecting abrupt terrain changes and edge noise.

### 4.4. Algorithm Robustness Analysis

**Figure 12:**
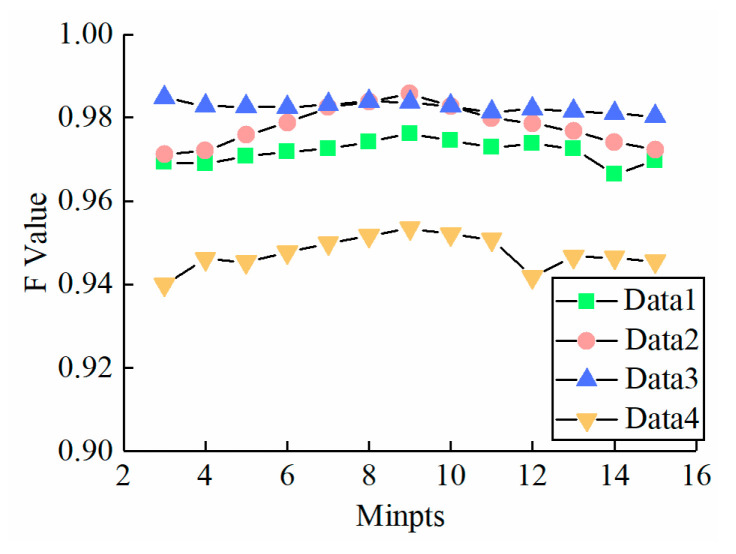
F value of MTANR for different input parameters of each test data.

To explore how the parameter MinPts affects MTANR’s performance, a series of MinPts were set in each dataset, and the *F* value in every input parameter is shown in Figure 12. As depicted in Figure 12, with the MinPts increasing from 3 to 15 at a step length of 1, it remains at an overall high level, although it fluctuates a little. For OPTICS-derived algorithms, though the *CD* values and *RD* values of photons may vary with different parameters, they change at roughly similar magnitudes, so there is no apparent variation in the order of photons in the *RD* list [24]. Therefore, the denoising results perform stably under different conditions.

### 4.5. Challenge under Extremely Dense Vegetation

**Figure 13:**
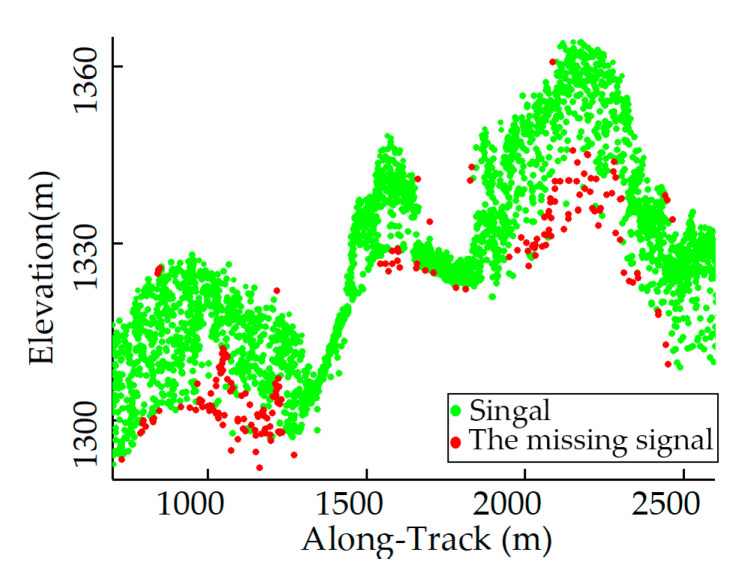
The missing signals under extremely dense vegetation.

From the experiment, we found that the proposed MTANR may misidentify near-ground photons for noise in regions with extremely dense canopy cover (e.g., the red point in Figure 13), which could lead to errors in ground elevation extraction. This is primarily caused by two factors. First, ATLAS is unable to penetrate dense canopies due to limitations in its detection mechanism and laser intensity [41]. As fraction vegetation coverage (FVC) increases, more photons will be reflected from the canopy, resulting in a reduction in the amount of ground-returned photons [16]. Secondly, density-based denoising methods remove noise by assuming that the spatial distribution of the signal is dense, whereas the noise distribution is relatively sparse. In this instance, the density of the sparse ground photons is comparable to that of the noise, making them readily misclassified [16,35,54]. Therefore, density clustering may not be appropriate in locations with dense canopies. Possible solutions [35,55,56] might be correcting and compensating the missing ground photons based on local ground continuity after edge searching.

## 5. Conclusions

This research presents a terrain-adaptive multilevel noise removal method (MTANR) with a coarse-to-fine denoising strategy. Preprocessing is achieved through histogram-based successive denoising to realize a coarse noise removal and provide an accurate range of signals to the maximum. In fine denoising, terrain-adaptive OPTICS and Otsu’s method were used to remove most of the sparse noise photons and create a near-noise-free environment for edge noise detection. Finally, edge searching was used to identify edge noise in residual photon clusters, and the signals were further maintained.

Based on the testing of experimental areas, we have drawn the following conclusions:

(1) The rotatably elliptical search domain can adapt to complex terrains and effectively utilize slope information. Compared to typical algorithms with fixed search domains, the proposed MTANR robustly retains sparse signals in steep areas and terrains with abrupt changes.
(2) Edge searching improves the ability to distinguish between signal and edge noise, which effectively improves denoising performance and minimizes the effects of edge noise in ICESat2 data applications like canopy height inversion.

In the future, we will investigate the ground-returned photon detection approach and denoising performance in dense vegetation.

## Figures and Tables

**Table 1:** The ICESat-2 data acquisition time and name details.

Areas	Datasets	Name	Time
Baishan	Data 1	ATL03_20210908041528_11691202_005_01	228.51~229.47 s
	Data 2	ATL03_20181021063658_03460102_005_02	233.25~233.52 s
New Zealand	Data 3	ATL03_20210502192018_05951109_005_01	5.50~6.99 s
	Data 4	ATL03_20210225222450_09761009_005_01	11.35~11.77 s

**Table 2:** Confusion metrics.

	Real	Signal	Noise
Predict			
Signal		*TP*	*FN*
Noise		*FP*	*TN*

**Table 3:** Evaluation of histogram-based successive denoising.

	Data 1	Data 2	Data 3	Data 4
*R_s_*	1	1	1	1
*R_n_*	0.8971	0.9235	0.873	0.8707

**Table 4:** Performance evaluation of ATL08, LDS, horizontal OPTICS, and MTANR.

Datasets		ATL08	LDS	Horizontal OPTICS	Terrain- Adaptive OPTICS	MTANR (Adaptive OPTICS +Edge Searching)
Data 1	*R*	0.9713	0.9987	0.9439	0.9978	0.9613
	*p*	0.9271	0.8446	0.9284	0.9397	0.9841
	*F*	0.9487	0.9152	0.9360	0.9638	0.9762
Data 2	*R*	0.9516	0.9929	0.9317	0.9853	0.9836
	*p*	0.9761	0.9613	0.9872	0.9755	0.9878
	*F*	0.9637	0.9768	0.9586	0.9803	0.9857
Data 3	*R*	0.8791	0.9843	0.9324	0.9984	0.9779
	*p*	0.9884	0.9303	0.9890	0.9623	0.9900
	*F*	0.9303	0.9565	0.9598	0.9807	0.9839
Data 4	*R*	0.8709	0.9615	0.8673	0.9352	0.9238
	*p*	0.9197	0.8831	0.9753	0.9521	0.9851
	*F*	0.8947	0.9207	0.9181	0.9436	0.9534

## Data Availability

The open data used in this article (access to the data is listed in the article) and the ICESat-2 data can be obtained by accessing the the Earthdata website (https://search.earthdata.nasa.gov, accessed on 4 March 2023), and the airborne data can be obtained by accessing the Toitū Te Whenua Land Information New Zealand website (https://data.linz.govt.nz, accessed on 18 March 2023).

## Appendix A

**Table A1:** The details of parameters used for matching ATL03 and ATL08.

Datasets	Parameters	Descriptions [17,18]
ATL03	signal_conf_ph	Confidence level associated with each photon event selected as the signal. (0 = noise, 1 = added to allow for buffering but the algorithm classifies it as background, 2 = low, 3 = med, and 4 = high.)
	segment_id	A 7-digit number identifies the along-track geolocation segment number.
ATL08	classed_pc_flag	Land Vegetation ATBD classification flag for each photon as either noise, ground, canopy, or top of canopy. (0 = noise, 1 = ground, 2 = canopy, and 3 = top of canopy.)
	classed_pc_indx	Index (1-based) of the ATL08 classified signal photon from the start of the ATL03 geolocation segment specified on the ATL08 product at the photon rate in the corresponding parameter, ph_segment_id. This index traces back to the specific photon within a 20 m segment_id on ATL03.
	ph_segment_id	Segment ID of photons tracing back to specific 20 m segment_id on ATL03.
